# Obesity and Breast Cancer: Current Insights on the Role of Fatty Acids and Lipid Metabolism in Promoting Breast Cancer Growth and Progression

**DOI:** 10.3389/fendo.2017.00293

**Published:** 2017-10-30

**Authors:** Christina Blücher, Sonja C. Stadler

**Affiliations:** ^1^Institute of Laboratory Medicine, Clinical Chemistry and Molecular Diagnostics, University Hospital Leipzig, Leipzig, Germany; ^2^LIFE – Leipzig Research Center for Civilization Diseases, Leipzig University, Leipzig, Germany

**Keywords:** breast cancer, obesity, adipose tissue, lipid metabolism, free fatty acids

## Abstract

Obesity and excess accumulation of adipose tissue are known risk factors for several types of cancer, including breast cancer. With the incidence of obesity constantly rising worldwide, understanding the molecular details of the interaction between adipose tissue and breast tumors, the most common tumors in women, becomes an urgent task. In terms of lipid metabolism, most of the studies conducted so far focused on upregulated *de novo* lipid synthesis in cancer cells. More recently, the use of extracellular lipids as source of energy came into focus. Especially in obesity, associated dysfunctional adipose tissue releases increased amounts of fatty acids, but also dietary lipids can be involved in promoting tumor growth and progression. In addition, it was shown that breast cancer cells and adipocytes, which are a major component of the stroma of breast tumors, are able to directly interact with each other. Breast cancer cells and adjacent adipocytes exchange molecules such as growth factors, chemokines, and interleukins in a reciprocal manner. Moreover, it was shown that breast cancer cells can access and utilize fatty acids produced by neighboring adipocytes. Thus adipocytes, and especially hypertrophic adipocytes, can act as providers of lipids, which can be used as a source of energy for fatty acid oxidation and as building blocks for tumor cell growth.

## Introduction

Breast cancer is the most abundant malignant tumor and the leading cause of death from cancer in women worldwide (1, 2). Established risk factors for breast cancer are a woman’s age, own or familial history of breast cancer or of precancerous lesions, genetic configuration, pregnancies and reproductive treatment, consumption of alcohol, and exposure to ionizing radiation (3). In addition, overweight and obesity are now regarded as promoting factors for breast cancer development and progression. This perception is based on numerous recent epidemiological and experimental studies with following observations: several population studies demonstrated that obesity and associated excess accumulation of adipose tissue are associated with an elevated risk for breast cancer, especially in post-menopausal women (4–6) and are independent negative prognostic factors for mammary tumors (7–10). On a molecular level, several studies showed that adipocytes, which are a major component of the stromal environment of mammary tumors, exert tumor-promoting effects on breast cancer cells. Several hypotheses about how adipose tissue and adipocytes promote tumorigenesis have been described, but the molecular mechanisms that underly this interaction are yet to be defined in more detail.

**Figure 1:**
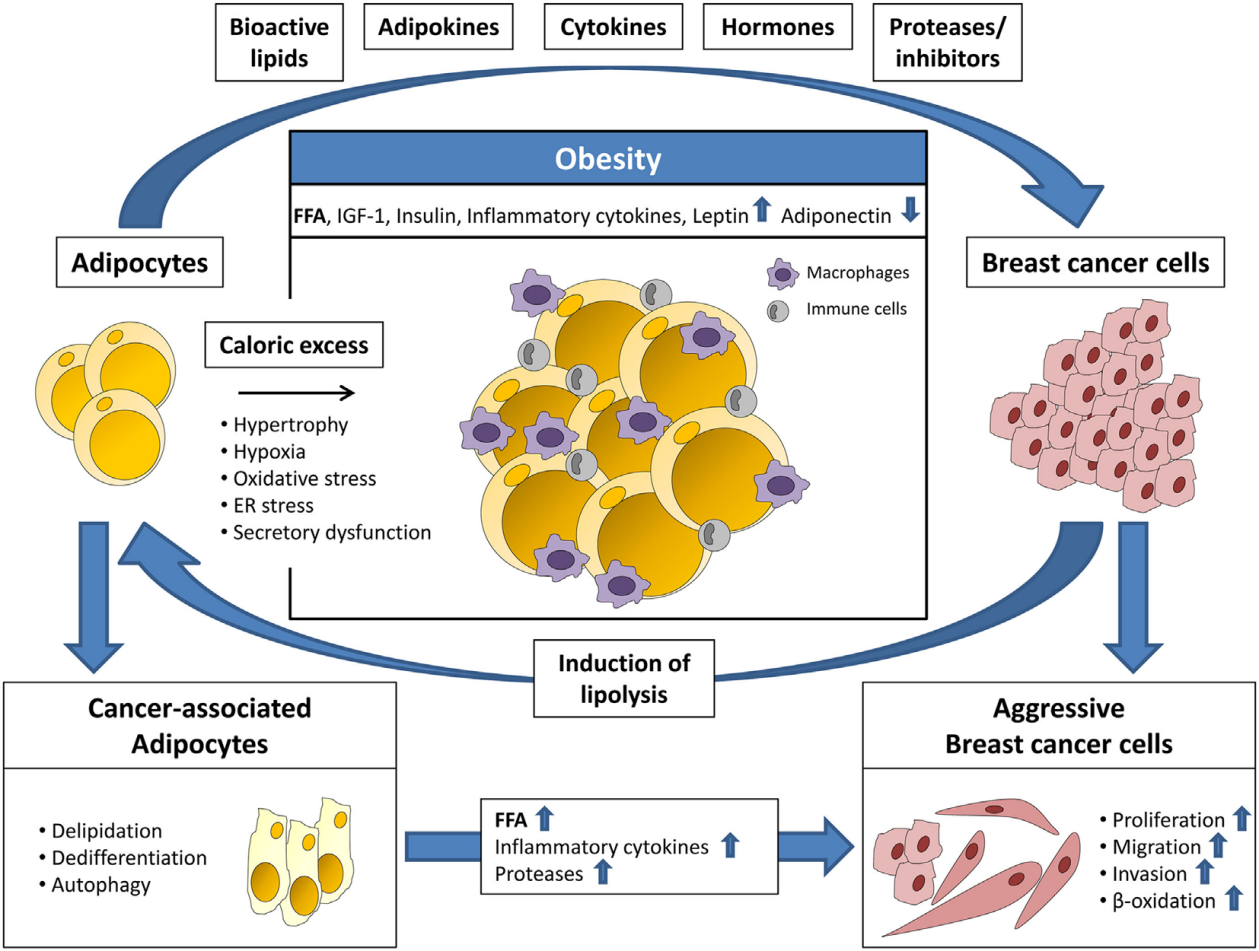
Adipocytes and breast cancer cells interact *via* several secreted factors. Adipocytes secrete bioactive lipids, adipokines, cytokines, hormones, and proteases/protease inhibitors priming breast cancer cells for a more aggressive phenotype. This includes increased proliferation, migration, invasion, and β-oxidation. Breast cancer cells induce adipocyte lipolysis resulting in the formation of cancer-associated adipocytes (CAAs), which are characterized by delipidation, dedifferentiation, autophagy, and altered secretion. In turn, the increased release of free fatty acids (FFA), inflammatory cytokines, and proteases from CAAs promotes breast cancer progression. In obesity, the adipose tissue is characterized by hypertrophy and increased infiltration of macrophages and other immune cells. Furthermore, adipocyte function is impaired due to hypoxia, oxidative, and ER stress leading to secretory dysfunction. The resulting elevated release of FFA, insulin-like growth factor-1 (IGF-1), insulin, inflammatory cytokines, and leptin, and decreased secretion of adiponectin, enhances the tumor-promoting effects of adipose tissue.

Signaling molecules and metabolites secreted by adipose tissue and adipocytes, especially in the obese state, are now recognized as important factors for cancer progression as they directly or indirectly stimulate anti-apoptotic effects, cell growth, angiogenesis, and migration (11, 12). Mature adipocytes are the major cell type of white adipose tissue and are primarily responsible for the metabolic homeostasis of the body. Lipids are stored here in the form of triacylglycerol (TAG) and released as free fatty acids (FFA) in times of demand. Besides energy storage, adipocytes also play an active role in endocrine signaling to other tissues of the body, by secreting hormones, adipokines, cytokines, and growth factors (13, 14). An elevated intake of calories and a largely sedentary lifestyle can lead to obesity, which often results in dysfunctional adipose tissue. In particular, adipocytes become hypertrophic and store elevated amounts of TAGs along with higher secretion of adipokines and pro-inflammatory cytokines, such as tumor necrosis factor-α, IL-6, IL-8, and PAI-1 (Figure 1). These molecules are chemoattractants for macrophages, monocytes, and other immune cells, which induce a chronic low-grade inflammation within the adipose tissue. As a result, lipolysis is initiated and adipocytes release elevated amounts of FFAs, which adversely affects lipid homeostasis of the entire organism and leads to subsequent metabolic diseases (12). The release of higher amounts of fatty acids could be a direct mechanism through which adiposity may promote cancer progression by delivering building blocks for the production of pro-tumorigenic signaling lipids (14).

Visceral obesity and increased adipose tissue mass are often accompanied by low levels of plasma high-density-lipoprotein cholesterol (HDL-C), which has been associated with breast cancer risk in some studies (15, 16). However, evidence for the relationship between plasma HDL-C and breast cancer risk remains equivocal (17) and it is not clear whether low HDL-C causally affects tumorigenesis or merely serves as a biomarker for poor lifestyle and dietary habits.

Epidemiologic studies have also investigated the relationship between elevated plasma low-density-lipoprotein cholesterol or total cholesterol and cancer occurrence and prognosis. In terms of breast cancer onset, these studies yielded contradictory findings (17, 18). However, in several recent studies, elevated plasma cholesterol levels were associated with a poor prognosis and the use of cholesterol-lowering medication (statins) increased recurrence-free survival of breast cancer patients (19–21). Large-scale prospective studies, adequately controlled for confounding factors, are necessary to substantiate the potential beneficial effects of statins and other cholesterol-lowering drugs in breast cancer patients.

Regarding intracellular lipid metabolism, it is well known that several tumor cells show a hyperactivation of various lipid synthesis pathways, including breast cancer. Breast cancer cells show an increased activity of fatty acid synthase (FASN), an enzyme used for *de novo* fatty acid synthesis. In addition, breast cancer cells also show an upregulation of monoacylglycerol lipase (MAGL). The MAGL pathway controls the intracellular release of fatty acids and its hyperactivation is often associated with the aggressiveness of a tumor. Together, FASN and MAGL very likely promote cancer progression by synthesizing and mobilizing intracellular lipids, which in turn promote tumor growth (14, 22, 23). Interestingly, lipidomic analyses demonstrated that the incorporation of endogenous fatty acids into membrane phospholipids is enhanced in mammary carcinomas as compared to normal human breast tissue. Furthermore, these changes in membrane lipid composition correlated with tumor progression, hormone receptor status, and patient survival, with the concentration of these lipids being the highest in ER-negative and grade 3 tumors (24). Moreover, another study showed that the ratios of specific monounsaturated fatty acid phosphatidylcholines compared to saturated fatty acid phosphatidylcholines are significantly higher in cancerous tissue in comparison to healthy reference sections (25). A different aspect of the role of lipid metabolism in cancer is seen in patients with late-stage cancers, who often suffer from cachexia. This phenomenon is characterized by the loss of both muscle and fat mass through catabolic mechanisms. This process is triggered by a marked upregulation of adipose triglyceride lipase (ATGL) and hormone-sensitive lipase (HSL), which break triglycerides into diglycerides and diglycerides into fatty acids, respectively. The resulting elevated levels of circulating FFA can be used as building blocks for cancer cell growth or tumorigenic signaling lipids (26). Thus, cancer cells are able to utilize FFA not just from *de novo* lipogenesis but also from exogenous fat sources. Intriguingly, a few recent articles described that breast cancer cells can access and directly use lipids from neighboring adipocytes (27, 28). One study even demonstrated that the predominant source of *de novo* lipid synthesis by breast cancer cells is extracellular lipids, not just glucose and glutamine (27). Together, these studies indicate that breast cancer cells are metabolically very flexible and fit the current notion that metabolic reprogramming is an emerging hallmark of cancer cells. However, in contrast to endocrine and paracrine effects of adipose tissue in obesity, the role of extracellular fatty acids in breast cancer metabolism is a relatively new area of research and warrants further elucidation.

In this review, we will focus on the role of lipids from excess adipose tissue in obesity, from tumor-associated adipocytes or dietary lipids, and discuss how these extracellular fatty acids drive tumor growth and progression.

## Lipids Delivered to Breast Cancer Cells Fuel Tumor Growth

### Direct Interaction of Adipocytes and Breast Cancer Cells

The tumor microenvironment plays an important role for its growth and progression since non-malignant cells of the stroma, such as endothelial cells, immune cells, tumor-associated macrophages and tumor-associated fibroblasts, deliver tumor-promoting molecules, including chemokines, interleukins, and growth factors (29). In breast cancer, the interaction of breast tumor cells with surrounding fibroblasts, immune, endothelial, and mesenchymal cells is well studied. By contrast, the crosstalk of breast tumor cells with associated adipocytes has been addressed only recently. In fact, adipocytes are a major component of the microenvironment of mammary tumors. During early tumor cell invasion, breast cancer cells invade the mammary fat pad and exist in direct conjunction with neighboring adipocytes (30). Several recent studies demonstrated that this direct interaction with adipocytes has tumor-promoting effects (Figure 1). Breast cancer cells secrete, among other factors, cytokines and lypolytic enzymes, which affect adipocytes. In a reciprocal manner, associated adipocytes secrete adipokines, growth factors, proteases, and fatty acids, which stimulate tumor growth and survival (31, 32). In addition, a study by Dirat et al. showed that breast cancer cells induce lipolysis together with a phenotypic change in neighboring adipocytes. These fat cells, termed cancer-associated adipocytes (CAAs), are characterized by a fibroblast-like morphology, a significant decrease in number and size of intracellular lipid droplets and loss of terminal adipocyte differentiation markers, such as leptin or FABP2 (Figure 1). Functionally, CAAs secrete increased amounts of proteases and interleukins, such as PAI-1, IL-6, and IL-1β, which promote tumor aggressiveness. In addition, CAAs were shown to deliver fatty acids, important building blocks for tumor proliferation (30). Using a co-culture model of ovarian cancer cells and omental adipocytes, Nieman and co-workers showed that cancer cells have the ability to take up and utilize fatty acids from surrounding fat cells (28). This co-cultivation induced lipolysis within the adipocytes and enabled a direct transfer of lipids to the cancer cells together with enhanced lipid storage and mitochondrial oxidation. Analogous co-cultivation of omental adipocytes with MCF-7 and MDA-MB-231 breast tumor cells also resulted in lipid droplet accumulation in the cancer cells (28). The impact of adipocyte-derived fatty acids on breast cancer cell progression was underscored by work conducted by Balaban et al. showing that MCF-7 and MDA-MB-231 breast cancer cells induced HSL/ATGL-dependent lypolysis in co-cultured adipocytes which resulted in increased cancer cell proliferation and migration. This effect was even more enhanced when adipocytes were loaded with a mixture of oleate, palmitate, and linoleate beforehand of co-cultivation to mimic “obese” adipocytes and thereby demonstrated that an increased availability of fatty acids for mitochondrial oxidation promotes breast cancer cell progression (27). Together, these studies suggest a metabolic shift of cancer cells in adaption to the availability of metabolic substrates in the microenvironment. This metabolic shift activates alternative pathways to support tumor growth and survival. To date, most of the studies examining breast cancer cell lipid metabolism focused on glucose and glutamine metabolism as precursors for *de novo* lipogenesis. The data described above clearly point out that extracellular lipids are an important source for breast cancer cell lipid synthesis and fatty acid oxidation. The translational relevance of these findings is substantiated by a recent study by Camarda et al. (33). The authors demonstrate that highly aggressive triple-negative breast cancer cells, which overexpress the oncogenic transcription factor MYC, show significantly increased rates of fatty acid oxidation. Pharmacological inhibition of fatty acid oxidation dramatically decreased energy metabolism and, therefore, cell and tumor growth *in vitro* and *in vivo* in a MYC-dependent manner. Together, these data highlight that targeting lipid metabolism and lipid uptake should be considered for the development of novel therapeutic strategies in breast cancer.

### Fatty Acids Released by Adipose Tissue in Obesity

Obesity is described as excess fat storage and accumulation of adipose tissue, which becomes deregulated. Dysfunctional adipocytes release increased amounts of fatty acids which accumulate in non-adipose tissues, such as liver, heart, or muscle. Intermediates of intracellular fatty acid metabolism, such as ceramides or diacylglycerols (DAGs), can ultimately induce lipotoxicity (34). Lipotoxicity is characterized by cell cycle and mitochondrial deregulation, autophagy, and apoptosis. Several recent studies have shown that an over-production of ceramides or DAGs induces growth arrest and apoptosis in various cancer cells (35, 36). These discoveries open interesting inroads for the development of new lipid-based cancer treatment options. On the other hand, elevated levels of fatty acids can be utilized by cancer cells as source of energy or as building blocks for oncogenic lipid signaling molecules, such as lysophosphatidic acid (LPA), prostaglandins and sphingosine-1-phosphate (S1P) (Figure 1) (14). In the past few years, several studies addressed the cellular and molecular mechanisms linking fatty acids and cancer using cell culture experiments and animal models. For example, oleate, which is the most abundant fatty acid esterified to triglycerides in adipose tissue, has been explored for its potential role in cancer progression (37–39). A recent *in vitro* study points in the direction that breast cancer cells use exogenous lipids, such as oleate, to regulate lipid metabolism, in addition to *de novo* fatty acid synthesis (40). Moreover, the authors show that a proliferative effect of oleate on breast cancer cells is dependent on the fatty acid translocase/CD36, as silencing of CD36 mRNA expression significantly decreased exogenous fatty acid uptake, which turns CD36 into an interesting candidate for novel treatment strategies (40). Also recently, Shen et al. demonstrated that oleate induces the expression of angiopoietin-like 4 (*ANGPTL4*) in head and neck squamous cell carcinoma resulting in anoikis resistance and metastasis *via* upregulation of fibronectin (41). Notably, palmitate and linoleate also induced *ANGPTL4* gene expression in these cancer cells. Moreover, the induction of *ANGPTL4* expression by oleate was also detected in other cancer cell types, including breast cancer cells (41). This suggests an interesting link since Angptl4 has been described to promote breast cancer cell invasion and metastasis to the lung *in vitro* and *in vivo*, respectively (42–44). The role of oleate was also studied with respect to metabolic adaptions in highly aggressive cancer cells. An *in vitro* study by Li and co-workers showed that AMPK is activated in highly metastatic gastric and breast cancer cells treated with oleate (45). AMPK promoted the rates of fatty acid oxidation and ATP synthesis in these cells, enabling increased cell growth and cell migration. In low metastatic cancer cells, oleate reduced cell proliferation and migration, indicating a selective tumor-promoting function of oleate on highly metastatic cancer cells (45). The pro-tumorigenic effect of oleate was also demonstrated by an independent study showing that the treatment with oleate promoted cell invasion in highly metastatic breast cancer cells, but not in low metastatic cancer cells (38). Addressing the potential underlying mechanism, Hardy et al. showed that oleate enhanced cell proliferation *via* activation of G protein-coupled receptor 40 in highly aggressive breast cancer cells (46). Moreover, oleate treatment of breast cancer cells resulted in long-term survival in serum-free media, which was associated with enhanced intracellular lipid droplet formation and upregulation of lipolysis (47). In contrast to the tumor-promoting effects of oleate, palmitate, which is the most abundant circulating saturated fatty acid in the human circulation, exhibited inhibitory effects in *in vitro* studies (48, 49). For example, the treatment of breast cancer cells with palmitate mediated the inhibition of cell proliferation and induction of apoptosis. Interestingly, oleate antagonized the proapoptotic function of palmitate in these experiments (49).

Together, these data indicate that the effects of fatty acids on breast cancer progression are complex and depend on the fatty acid subtype, the combination thereof, and the specific breast cancer subtype. More future studies are warranted to uncover the detailed link between obesity, fatty acids, fatty acid metabolism intermediates, and breast cancer progression.

### Cholesterol Metabolism and Breast Cancer

Changes in cholesterol and lipid metabolism (often due to poor diet or obesity) have been extensively studied as risk factors for various malignancies, including breast cancer. Several epidemiological studies investigated the relationship between cholesterol and the risk of breast cancer, with inconsistent results (17). However, Li and co-workers demonstrated in a more recent meta-analysis study that dietary cholesterol was associated with an increased risk of breast cancer (50). Evidence for the role of elevated plasma cholesterol in promoting breast cancer was also obtained in recent experimental studies. The induction of hypercholesterolemia in mice resulted in enhanced breast cancer growth, suggesting tumor-promoting effects of hypercholesterolemia (51, 52). Moreover, the primary oxysterol metabolite of cholesterol, 27-hydroxycholesterol (27-OHC), was identified to promote growth and metastasis *in vivo* (53, 54). Higher levels of 27-OHC were also detected in human estrogen receptor-positive breast tumors as compared to adjacent normal breast tissue (55). In addition, 27-OHC was also described to play a crucial role in mediating resistance of estrogen receptor-positive breast cancer to specific endocrine therapies (56, 57). Together the data show that alterations in lipid and cholesterol metabolism might be important factors in promoting breast cancer progression. To fully understand how obesity and associated changes in lipid metabolism affect breast cancer biology is going to be one of the demanding but irremissible tasks in battling breast cancer.

### The Role of Omega-3 and Omega-6 Polyunsaturated Fatty Acids (PUFAs) in Breast Cancer

The impact of FFA and specific components, such as saturated, monounsaturated, and PUFAs, were studied in several human diseases, including cancer. Much of the data implicate that saturated fatty acids and monounsaturated fatty acids elevate cancer risk, whereas specific PUFAs (omega-3 PUFAs) exhibit anticancer effects (58–61). Still, since not all of the studies conducted so far showed consistent results, more detailed analyses are warranted. Particularly with regard to breast cancer, the contribution of dietary fatty acids depends on diverse factors, e.g., breast cancer subtype, a woman’s menopausal status, fatty acid species, and intake ratios (62).

The two major groups of PUFAs, omega-3 and omega-6 PUFAs, are essential fatty acids, which must be ingested as part of a diet. Omega-3 PUFAs, such as eicosapentaenoic acid and docosahexaenoic acid are precursors for the production of anti-inflammatory eicosanoids and inflammation resolving derivates, such as resolvins and protectins (63). On the other hand, eicosanoids resulting from the omega-6 PUFA–arachidonic acid (AA) axis are predominantly involved in the initiation and maintenance of inflammation (63). In recent years, epidemiologic studies have explored the role of omega-3 and omega-6 PUFAs on cancer risk and reported that consumption of western diets with a low omega-3:omega-6 ratio is associated with a higher risk of several cancer types (64). Notably, an elevated intake of omega-3 PUFAs as well as a higher dietary intake ratio of omega-3:omega-6 PUFAs correlated with reduced breast cancer risk in obese women, but there was no such association in overweight or normal weight women (65). Thus, this study suggests a link between obesity, omega-3-PUFAs intake, and breast cancer risk. Several mechanisms have been proposed for the anti-tumor effects of omega-3 PUFAs, including the alteration of the cell plasma membrane composition, the inhibition of AA-derived synthesis of inflammatory eicosanoids, and alteration of gene expression of genes known to be involved in cell proliferation and apoptosis (62). Especially in connection with obesity, omega-3 PUFAs might be a useful tool in reducing obesity-associated inflammation and related tumor risk (66, 67).

In conclusion, these studies support the interesting notion that PUFAs, especially omega-3 PUFAs, are linked to reduced breast cancer risk, in particular by decreasing pro-tumorigenic inflammation. However, more clinical studies are needed to fully understand the role of omega-3 and omega-6 PUFAs in obesity-associated breast cancer.

## Summary

Obesity is now recognized as an important risk factor for breast cancer development and progression. Several mechanisms have been suggested to explain this association, including inflammatory signaling, chemokines, adipokines, and insulin. In addition, more recent studies demonstrated that extracellular lipids play an important role in promoting breast cancer growth and progression by serving as substrates for activated fatty acid oxidation or as building blocks for oncogenic lipid signaling molecules. Breast cancer cells may obtain extracellular lipids through deregulated adipose tissue, by dietary intake or by directly interacting with adipocytes of the tumoral stroma. Emerging evidence clearly indicates that breast tumor cells are able to adapt to their metabolic environment in a very flexible manner. Targeting the utilization of extracellular lipids in breast tumor cells may open up new avenues for breast cancer treatment.

## Author Contributions

CB and SCS conceived the review, critically analyzed the current literature, and wrote and revised the manuscript.

## Conflict of Interest Statement

The authors declare that the research was conducted in the absence of any commercial or financial relationships that could be construed as a potential conflict of interest.
